# The effect of cangrelor and access site on ischaemic and bleeding events: insights from CHAMPION PHOENIX

**DOI:** 10.1093/eurheartj/ehv498

**Published:** 2015-09-23

**Authors:** J. Antonio Gutierrez, Robert A. Harrington, James C. Blankenship, Gregg W. Stone, Ph. Gabriel Steg, C. Michael Gibson, Christian W. Hamm, Matthew J. Price, Philippe Généreux, Jayne Prats, Efthymios N. Deliargyris, Kenneth W. Mahaffey, Harvey D. White, Deepak L. Bhatt, on behalf of the CHAMPION PHOENIX Investigators, DL Bhatt, GW Stone, KW Mahaffey, CM Gibson, PG Steg, CW Hamm, MJ Price, S Leonardi, D Gallup, E Bramucci, PW Radke, P Widimsky, F Tousek, J Tauth, D Spriggs, BT McLaurin, DJ Angiolillo, P Genereux, T Liu, J Prats, M Todd, S Skerjanec, HD White, RA Harrington

**Affiliations:** 1 Brigham and Women's Hospital Heart & Vascular Center and Harvard Medical School, 75 Francis Street, Boston, MA 02115, USA; 2 Stanford University Medical School, Stanford, CA, USA; 3 Geisinger Medical Center, Danville, PA, USA; 4 Columbia University Medical Center and the Cardiovascular Research Foundation, New York, NY, USA; 5 DHU FIRE, INSERM Unité 1148, Université Paris-Diderot, and Hôpital Bichat, Assistance-Publique–Hôpitaux de Paris, Paris, France; 6 NHLI, Imperial College, Royal Brompton Hospital, London, UK; 7 Beth Israel Deaconess Medical Center, Division of Cardiology, Boston, MA, USA; 8 Kerckhoff Heart and Thorax Center, Bad Nauheim, Germany; 9 Scripps Clinic and Scripps Translational Science Institute, La Jolla, CA, USA; 10 The Medicines Company, Parsippany, NJ, USA; 11 Green Lane Cardiovascular Service, Auckland, New Zealand

**Keywords:** Percutaneous coronary intervention, Platelet inhibition, Radial artery

## Abstract

**Aims:**

To assess whether the use of the femoral or radial approach for percutaneous coronary intervention (PCI) interacted with the efficacy and safety of cangrelor, an intravenous P2Y_12_ inhibitor, in CHAMPION PHOENIX.

**Methods and results:**

A total of 11 145 patients were randomly assigned in a double-dummy, double-blind manner either to a cangrelor bolus and 2-h infusion or to clopidogrel at the time of PCI. The primary endpoint, a composite of death, myocardial infarction, ischaemia-driven revascularization, or stent thrombosis, and the primary safety endpoint, Global Use of Strategies to Open Occluded Coronary Arteries (GUSTO) defined severe bleeding, were evaluated at 48 h. Of the patients undergoing PCI and receiving study drug treatment, a total of 8064 (74%) and 2855 (26%) patients underwent femoral or radial PCI, respectively. Among the femoral cohort, the primary endpoint rate was 4.8% with cangrelor vs. 6.0% with clopidogrel (odds ratio, OR [95% confidence interval, CI] = 0.79 [0.65–0.96]); among the radial cohort, the primary endpoint was 4.4% with cangrelor vs. 5.7% with clopidogrel (OR [95% CI] = 0.76 [0.54–1.06]), *P*-interaction 0.83. The rate of GUSTO severe bleeding in the femoral cohort was 0.2% with cangrelor vs. 0.1% with clopidogrel (OR [95% CI] = 1.73 [0.51–5.93]). Among the radial cohort, the rate of GUSTO severe bleeding was 0.1% with cangrelor vs. 0.1% with clopidogrel (OR [95% CI] = 1.02 [0.14–7.28]), *P*-interaction 0.65. The evaluation of safety endpoints with the more sensitive ACUITY-defined bleeding found major bleeding in the femoral cohort to be 5.2% with cangrelor vs. 3.1% with clopidogrel (OR [95% CI] = 1.69 [1.35–2.12]); among the radial cohort the rate of ACUITY major bleeding was 1.5% with cangrelor vs. 0.7% with clopidogrel (OR [95% CI] = 2.17 [1.02–4.62], *P*-interaction 0.54).

**Conclusion:**

In CHAMPION PHOENIX, cangrelor reduced ischaemic events with no significant increase in GUSTO-defined severe bleeding. The absolute rates of bleeding, regardless of the definition, tended to be lower when PCI was performed via the radial artery.

**Clinical trial registration:**

http://www.clinicaltrials.gov identifier: NCT01156571.

**See page 1131 for the editorial comment on this article (doi:10.1093/eurheartj/ehv570)**

## Introduction

The association between bleeding and increased morbidity and mortality after percutaneous coronary intervention (PCI) has prompted the implementation of bleeding reduction strategies at the time of PCI.^1,2^ According to clinical trial data, compared with femoral access, the radial artery approach for PCI has been demonstrated to have similar efficacy with the benefit of reduced major bleeding and access site complications.^3–6^ Owing to a favourable safety profile, several guidelines endorse the radial artery as the preferred PCI access site.^7,8^

Cangrelor is an intravenous P2Y_12_ receptor antagonist with an immediate (within 2 min) onset of action and a half-life of 3–6 min, allowing platelet function to return to baseline within 60 min of infusion cessation. In the Cangrelor vs. Standard Therapy to Achieve Optimal Management of Platelet Inhibition (CHAMPION) PHOENIX trial, the intravenous P2Y_12_ receptor antagonist, cangrelor, reduced the rate of ischaemic events at 48 h in patients undergoing percutaneous revascularization without a significant increase in severe bleeding or transfusions.^9^ In this pre-specified secondary analysis, we explore the efficacy and safety of cangrelor according to access site (femoral vs. radial) in the CHAMPION PHOENIX trial.

## Methods

### Patient population

**Figure 1:**
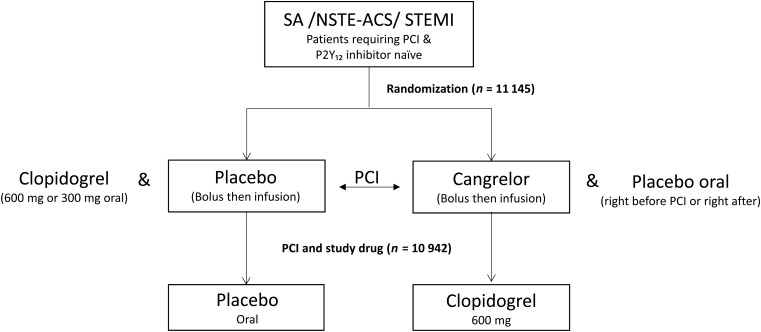
Study design. SA, stable angina; NSTE-ACS, non-ST segment elevation acute coronary syndrome; STEMI, ST segment elevation myocardial infarction; PCI, percutaneous coronary intervention.

CHAMPION PHOENIX was a double-blind, double-dummy, placebo-controlled trial of cangrelor in patients undergoing PCI. Both the design and primary findings have been published previously.^9,10^ Briefly, 11 145 patients undergoing either elective or urgent PCI and receiving guideline-recommended therapy were randomized after angiography to receive a bolus (30 µg/kg) and infusion (4 µg/kg/min for a minimum of 2 h or the duration of the procedure whichever was longer) of cangrelor or a 600 or 300 mg loading dose of clopidogrel. The timing (before or after PCI) and dose of clopidogrel were at the discretion of the site investigator. The access approach for PCI was determined by the site investigator and did not require institutional review board (IRB) approval. At the end of the infusion, patients then received either 600 mg of clopidogrel (cangrelor group) or matching placebo (clopidogrel group; *Figure 1*).

### Endpoints

The primary efficacy endpoint was a composite of death (by any cause), myocardial infarction (MI), ischaemia-driven revascularization (IDR), or stent thrombosis in the first 48 h following randomization. Criteria for MI within 48 h post-PCI were defined as an elevation in creatine kinase-myocardial band (CK-MB) greater than three times the upper limit of normal or by a combination of CK-MB elevation in addition to ischaemic symptoms, angiographic evidence, and/or ECG changes. The key secondary endpoint was the incidence of stent thrombosis at 48 h, which was defined according to the Academic Research Consortium criteria or as intraprocedural stent thrombosis.^11^ All events of death, MI, IDR, and stent thrombosis were adjudicated. The primary safety endpoint was severe non-coronary artery bypass grafting (CABG) bleeding according to the Global Use of Strategies to Open Occluded Coronary Arteries (GUSTO) criteria. Thrombolysis In Myocardial Infarction (TIMI) and Acute Catheterization and Urgent Intervention Triage strategY (ACUITY) bleeding definitions were also evaluated. Bleeding endpoints, based on pre-specified criteria, were derived from investigator-reported data using a computer algorithm. Bleeding endpoints were not adjudicated.

### Statistical analysis

This was a pre-specified analysis outlined in the CHAMPION PHOENIX study protocol, which was approved by local ethics committees. Independent verification of these analyses by Harvard Clinical Research Institute did not require specific IRB approval. Efficacy analyses were performed from the modified intention-to-treat (mITT) population, defined as subjects undergoing PCI and receiving study drug. Bleeding analyses were performed from the safety population, defined as subjects receiving study drug. Baseline characteristics were summarized by vascular access (femoral vs. radial) and treatment (cangrelor vs. clopidogrel); and were compared using analysis of variance for continuous variables and the *χ*^2^ test for categorical variables. Treatment comparisons within access site (cangrelor vs. clopidogrel) were not adjusted for baseline characteristics, were based on the *χ*^2^ test, and are presented as odds ratios with 95% confidence intervals (CIs). The number needed to treat (NNT) was derived within the femoral and radial subgroups as the inverse of the difference between the cangrelor and clopidogrel event rates. The interaction between treatment and PCI access site was tested using the Breslow–Day method. Time-to-event curves for the primary efficacy endpoint at 48 h were constructed using the Kaplan–Meier method and compared using the log-rank test.

Multivariable logistic regression modelling was performed using propensity scores to assess the effect of PCI access site (femoral or radial), using femoral approach as the reference, with study treatment in the model and the propensity score based on the following potential variables: diagnosis at presentation, region (US/non-US), smoking status, hyperlipidaemia, previous MI, prior PCI, prior CABG, peripheral artery disease, planned clopidogrel loading dose (300 or 600 mg), and anticoagulant (bivalirudin or heparin). Statistical analyses were conducted using the SAS software, version 9.3 (SAS Institute, Cary, North Carolina, USA).

## Results

### Patients

Out of 11 145 patients randomized in CHAMPION PHOENIX, 10 942 patients comprised the mITT population who received study treatment and underwent PCI. Of these patients, 8064 (74%) underwent PCI via the femoral artery and 2855 (26%) via the radial approach. Three patients in the radial cohort did not complete the 48-h post-PCI follow-up and were excluded from the efficacy endpoint analyses; 23 patients underwent brachial PCI and were not included in this analysis.

### Cangrelor vs. clopidogrel

Baseline characteristics according to access site (femoral vs. radial) and randomized treatment (cangrelor vs. clopidogrel) are depicted in *Table 1*. In the femoral cohort, subjects randomized to cangrelor had higher rates of peripheral artery disease (8.2 vs. 6.7%, *P* = 0.01). In the radial cohort, subjects randomized to cangrelor had a lower median weight (84 vs. 85 kg, *P* = 0.008).

**Table 1:** Baseline characteristics

	Femoral			Radial		
	Cangrelor	Clopidogrel	*P*-value	Cangrelor	Clopidogrel	*P*-value
Characteristic						
Demographic						
*n*	4053	4011		1410	1445	
Age, years						
Median	64	64	0.51	64.5	64	0.11
Interquartile range	56, 72	56, 72		57, 72	56, 71	
Female sex, *n* (%)	1144 (28.2)	1107 (27.6)	0.53	413 (29.3)	382 (26.4)	0.09
Weight, kg						
Median	84	84	0.86	84	85	0.008
Interquartile range	73, 96	73, 96		73, 95	75, 96	
Medical history, *n* (%)						
Diabetes mellitus	1106/4048 (27.3)	1094/4005 (27.3)	1.00	409/1407 (29.1)	438/1444 (30.3)	0.46
Current smoker	1142/3950 (28.9)	1148/3918 (29.3)	0.70	357/1380 (25.9)	398/1408 (28.3)	0.15
Hypertension	3239/4040 (80.2)	3156/3998 (78.9)	0.17	1127/1410 (79.9)	1164/1442 (80.7)	0.59
Hyperlipidaemia	2434/3507 (69.4)	2366/3447 (68.6)	0.49	923/1336 (69.1)	963/1376 (70.0)	0.61
Prior stroke or TIA	210/4039 (5.2)	180/3996 (4.5)	0.15	61/1407 (4.3)	61/1442 (4.2)	0.89
Prior myocardial infarction	844/4030 (20.9)	914/3982 (23.0)	0.03	245/1402 (17.5)	258/1435 (18.0)	0.73
Prior PTCA or PCI	913/4045 (22.6)	947/4003 (23.7)	0.25	353/1408 (25.1)	383/1444 (26.5)	0.38
CABG	495/4048 (12.2)	436/4006 (10.9)	0.06	81/1409 (5.7)	63/1444 (4.4)	0.09
Heart failure	412/4044 (10.2)	443/4002 (11.1)	0.20	137/1407 (9.7)	140/1440 (9.7)	0.99
Peripheral artery disease	328/4015 (8.2)	268/3978 (6.7)	0.01	119/1384 (8.6)	112/1427 (7.8)	0.47

TIA, transient ischaemic attack; PTCA, percutaneous transluminal coronary angioplasty; PCI, percutaneous coronary intervention; CABG, coronary artery bypass graft.

### Radial vs. femoral

Baseline characteristics according to access site only (femoral vs. radial) are depicted in the Supplementary material online, *Table S1*. Compared with the femoral cohort, subjects in the radial cohort had higher rates of diabetes mellitus (27.3 vs. 29.7%, *P* = 0.01) and prior PCI (23.1 vs. 25.8%, *P* = 0.004), but lower rates of current smoking (29.1 vs. 27.1%, *P* = 0.04), prior MI (21.9 vs. 17.7%, *P* < 0.0001), and prior CABG (11.6 vs. 5.0%, *P* < 0.0001) (Supplementary material online, *Table S1*).

### Procedure characteristics

#### Cangrelor vs. clopidogrel

Procedure characteristics according to access site (femoral vs. radial) and randomized treatment (cangrelor vs. clopidogrel) are depicted in *Table 2*. In the femoral cohort, subjects randomized to cangrelor had lower rates of glycoprotein IIb/IIIa inhibitor use (2.7 vs. 4.3%, *P* = 0.0001).

**Table 2:** Procedure characteristics

	Femoral			Radial		
	Cangrelor	Clopidogrel	*P*-value	Cangrelor	Clopidogrel	*P*-value
Indication, *n* (%)			0.95			0.06
Stable angina	2290/4053 (56.5)	2258/4011 (56.3)		888/1410 (63.0)	905/1445 (62.6)	
NSTE-ACS	1099/4053 (27.1)	1085/4011 (27.1)		365/1410 (25.9)	341/1445 (23.6)	
STEMI	664/4053 (16.4)	668/4011 (16.7)		157/1410 (11.1)	199/1445 (13.8)	
Antithrombotic, *n* (%)						
Aspirin	3851/4050 (95.1)	3796/4007 (94.7)	0.47	1304/1410 (92.5)	1338/1444 (92.7)	0.86
Clopidogrel, 300 mg loading dose (planned)	1322/4053 (32.6)	1332/4011 (33.2)	0.57	79/1410 (5.6)	62/1445 (4.3)	0.11
Clopidogrel, 600 mg loading dose (planned)	2731/4053 (67.4)	2679/4011 (66.8)	0.57	1331/1410 (94.4)	1383/1445 (95.7)	0.11
Low molecular weight heparin	580/4053 (14.3)	579/4011 (14.4)	0.87	150/1410 (10.6)	174/1443 (12.1)	0.23
Unfractionated heparin	3045/4053 (75.1)	3020/4010 (75.3)	0.85	1220/1410 (86.5)	1243/1445 (86.0)	0.70
Fondaparinux	117/4053 (2.9)	92/4011 (2.3)	0.09	39/1409 (2.8)	43/1445 (3.0)	0.74
Bivalirudin	944/4053 (23.3)	940/4009 (23.4)	0.87	307/1410 (21.8)	326/1445 (22.6)	0.61
Glycoprotein IIb/IIIa inhibitor	111/4053 (2.7)	173/4011 (4.3)	0.0001	41/1410 (2.9)	54/1445 (3.7)	0.22

NSTE-ACS, non-ST-elevation acute coronary syndrome; STE ACS, ST-elevation acute coronary syndrome; PCI, percutaneous coronary intervention.

#### Femoral vs. radial

Procedure characteristics according to access site only (femoral vs. radial) are depicted in the Supplementary material online, *Table S2*. Patients in the radial cohort had lower rates of aspirin (94.9 vs. 92.6%, *P* < 0.0001) and low molecular weight heparin (14.4 vs. 11.4%, *P* < 0.0001) use, but had higher rates of unfractionated heparin (75.2 vs. 86.3%, *P* < 0.0001) use at the time of PCI compared with patients in the femoral cohort. The clopidogrel 600 mg loading dose was used less often in the femoral (67.1%) cohort compared with the radial (95.1%) cohort, *P* < 0.0001. Percutaneous coronary intervention duration (17 min femoral vs. 18 min radial, *P* = 0.34) and success rate (98.2% femoral and 98.3% radial, *P* = 0.68) were similar in both groups. Of the femoral cohort, 53.5% of patients received a drug eluting stent, compared with 61.8% of patients in the radial cohort, *P* < 0.001.

### Outcomes

#### Cangrelor vs. clopidogrel

**Figure 2:**
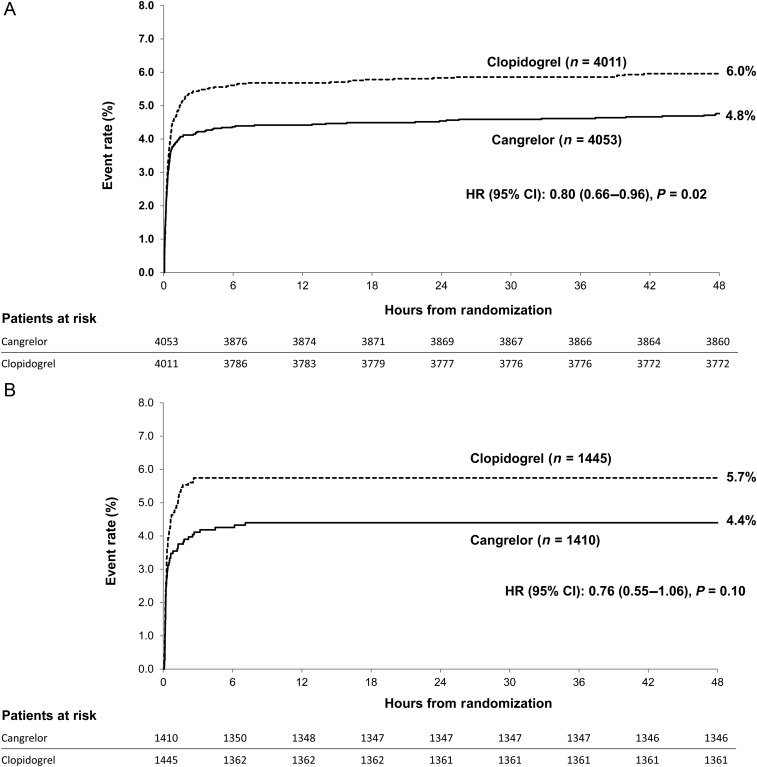
(*A*) Kaplan–Meier curves for the primary efficacy endpoint in the subgroup undergoing femoral access (cangrelor vs. clopidogrel). (*B*) Kaplan–Meier curves for the primary efficacy endpoint in the subgroup undergoing radial access (cangrelor vs. clopidogrel). HR, hazard ratio; CI, confidence interval.

**Figure 3:**
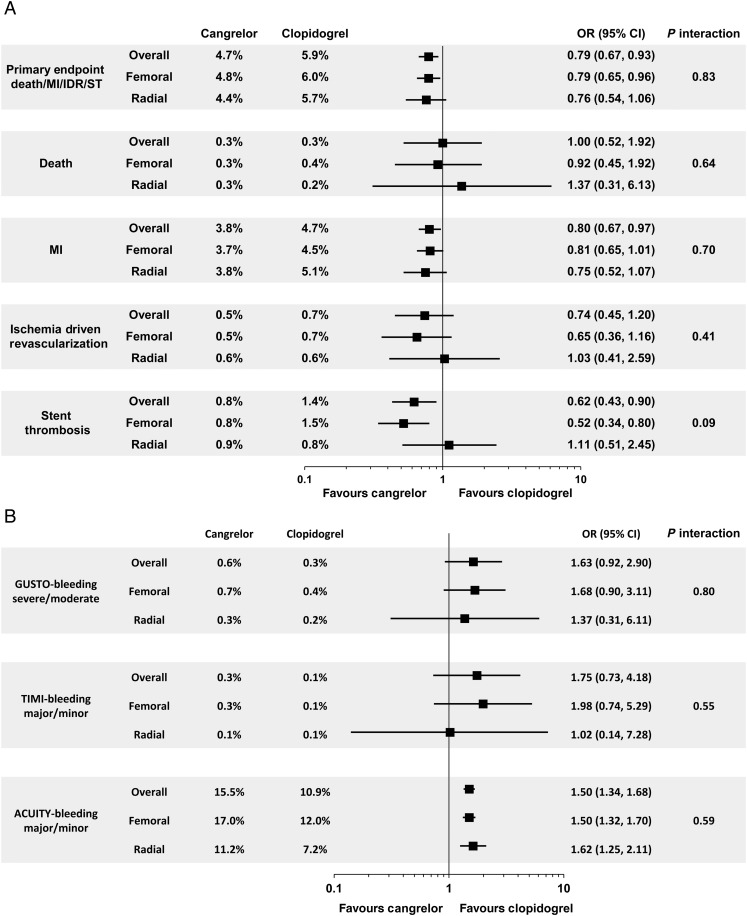
(*A*) Efficacy of cangrelor vs. clopidogrel in the femoral and radial subgroups. MI, myocardial infarction; IDR, ischaemia-driven revascularization; ST, stent thrombosis; OR, odds ratio; CI, confidence interval. (*B*) Bleeding with cangrelor vs. clopidogrel in the femoral and radial subgroups. GUSTO, Global Use of Strategies to Open Occluded Arteries; TIMI, Thrombolysis In Myocardial Infarction; ACUITY, Acute Catheterization and Urgent Intervention Triage strategY; OR, odds ratio; CI, confidence interval.

In the femoral cohort, the rate of the primary efficacy endpoint of death, MI, IDR, or stent thrombosis at 48 h was 4.8% with cangrelor vs. 6.0% with clopidogrel (odds ratio [OR] 95% CI = 0.79 [0.65–0.96]), NNT 84; in the radial cohort, the primary efficacy endpoint rate was 4.4% with cangrelor vs. 5.7% with clopidogrel (OR [95% CI] = 0.76 [0.54–1.06]), *P*-interaction = 0.83; NNT 74. *Figure 2A* and *B* depicts the Kaplan–Meier estimates for the time-to-event for the primary endpoint in both the femoral and radial cohorts. Among the femoral cohorts, the key secondary endpoint of stent thrombosis at 48 h was 0.8% with cangrelor vs. 1.5% with clopidogrel (OR [95% CI] = 0.52 [0.34–0.80]); in the radial cohort, the rate of stent thrombosis at 48 h was 0.9% with cangrelor vs. 0.8% with clopidogrel (OR [95% CI] = 1.11 [0.51–2.45]), *P*-interaction 0.09. The effects of cangrelor on the primary and secondary endpoints in the overall study population and according to access site are shown in *Figure 3A*.

In both access cohorts, there were no significant differences in the rates of GUSTO severe bleeding, TIMI major bleeding, or blood transfusions in patients treated with cangrelor compared with clopidogrel. In the femoral cohort, the rate of ACUITY major bleeding was 5.2% with cangrelor vs. 3.1% with clopidogrel (OR [95% CI] = 1.69 [1.35–2.12), *P* < 0.0001; in the radial cohort, the rate of ACUITY major bleeding was 1.5% with cangrelor vs. 0.7% with clopidogrel (OR [95% CI] = 2.17 [1.02–4.62]), *P* = 0.04 and *P*-interaction 0.54. The effects of cangrelor on bleeding according to access site and categorized by the various bleeding criteria are listed in *Table 3*. The effects of cangrelor on bleeding in the overall study population and according to access site are shown in *Figure 3B*.

**Table 3:** Safety endpoints at 48 h after randomization

Endpoint	Femoral				Radial				*P-*interaction
	Cangrelor	Clopidogrel	OR (95% CI)	*P*-value	Cangrelor	Clopidogrel	OR (95% CI)	*P*-value	
GUSTO bleeding									
Severe or life threatening	7/4052 (0.2)	4/4012 (0.1)	1.73 (0.51–5.93)	0.37	2/1411 (0.1)	2/1444 (0.1)	1.02 (0.14–7.28)	0.98	0.65
Moderate	20/4052 (0.5)	12/4012 (0.3)	1.65 (0.81–3.39)	0.16	2/1411 (0.1)	1/1444 (0.1)	2.05 (0.19–22.61)	0.55	0.87
Severe/moderate	27/4052 (0.7)	16/4012 (0.4)	1.68 (0.90–3.11)	0.10	4/1411 (0.3)	3/1444 (0.2)	1.37 (0.31–6.11)	0.68	0.80
TIMI bleeding									
Major	3/4052 (0.1)	3/4012 (0.1)	0.99 (0.20–4.91)	0.99	2/1411 (0.1)	2/ 1444 (0.1)	1.02 (0.14–7.28)	0.98	0.98
Minor	9/4052 (0.2)	3/4012 (0.1)	2.97 (0.80–11.00)	0.09	0/1411 (0.0)	0/1444 (0.0)			
Major/minor	12/4052 (0.3)	6/4012 (0.1)	1.98 (0.74–5.29)	0.16	2/1411 (0.1)	2/1444 (0.1)	1.02 (0.14–7.28)	0.98	0.55
ACUITY bleeding									
Major	209/4052 (5.2)	125/4012 (3.1)	1.69 (1.35–2.12)	<0.0001	21/1411 (1.5)	10/1444 (0.7)	2.17 (1.02–4.62)	0.04	0.54
Minor	510/4052 (12.6)	369/4012 (9.2)	1.42 (1.23–1.64)	<0.0001	139/1411 (9.9)	94/1444 (6.5)	1.57 (1.19–2.06)	0.001	0.53
Major/minor	690/4052 (17.0)	483/4012 (12.0)	1.50 (1.32–1.70)	<0.0001	158/1411 (11.2)	104/1444 (7.2)	1.62 (1.25–2.11)	0.0002	0.59
Blood transfusion	22/4052 (0.5)	14/4012 (0.3)	1.56 (0.80–3.05)	0.19	3/1411 (0.2)	2/1444 (0.1)	1.54 (0.26–9.21)	0.63	0.99

GUSTO, Global Use of Strategies to Open Occluded Arteries; Thrombolysis In Myocardial Infarction; ACUITY, Acute Catheterization and Urgent Intervention Triage strategY; OR, odds ratio; CI, confidence interval.

#### Femoral vs. radial

Among patients undergoing PCI via the femoral artery, the rate of the primary efficacy endpoint at 48 h was 5.4 vs. 5.1% in the radial group (unadjusted OR 95% CI = 0.95 [0.78–1.15]), *P* = 0.58. After multivariable analysis, compared with patients undergoing PCI via the femoral artery, there was no difference in the adjusted rate of primary efficacy endpoint in the radial access cohort (OR [95% CI] = 1.03 [0.81–1.29]), *P* = 0.83. Efficacy endpoints (unadjusted and adjusted) according to access site are displayed in Supplementary material online, *Table S3*.

In the femoral cohort, the rate of GUSTO severe/moderate bleeding was 0.5% compared with 0.2% in the radial cohort (unadjusted OR [95% CI] = 0.46 [0.21–1.02]; *P* = 0.05). The rate of TIMI major/minor bleeding in the femoral cohort was 0.2% compared with 0.1% in the radial cohort (unadjusted OR [95% CI] = 0.63 [0.21–1.85]; *P* = 0.39). Lastly, the rate of ACUITY major/minor bleeding was 14.5% in the femoral cohort and 9.2% in the radial group (unadjusted OR [95% CI] = 0.60 [0.52–0.68]; *P* < 0.0001). After multivariable analysis, compared with PCI via the femoral artery, using the radial approach was associated with a decreased odds of GUSTO severe/moderate (OR [95% CI] = 0.35 [0.12–1.01]; *P* = 0.05), TIMI major/minor bleeding (OR [95% CI] = 0.34 [0.07–1.54]; *P* = 0.16), and ACUITY major/minor bleeding (OR [95% CI] = 0.70 [0.59–0.83]; *P* < 0.0001 (*Table 4*).

**Table 4:** Safety endpoints at 48 h: radial vs. femoral

Endpoint	Femoral	Radial	OR (95% CI) unadjusted	*P*-value	OR (95% CI) adjusted	*P*-value
GUSTO bleeding						
Severe or life threatening	11/8064 (0.1)	4/2855 (0.1)	1.03 (0.33, 3.23)	0.96		
Moderate	32/8064 (0.4)	3/2855 (0.1)	0.26 (0.08, 0.86)	0.02		
Severe/moderate	43/8064 (0.5)	7/2855 (0.2)	0.46 (0.21, 1.02)	0.05	0.35 (0.12–1.01)	0.05
TIMI bleeding						
Major	6/8064 (0.1)	4/2855 (0.1)	1.89 (0.53, 6.67)	0.32		
Minor	12/8064 (0.1)	0/2855 (0.0)	–	0.04		
Major/minor	18/8064 (0.2)	4/2855 (0.1)	0.63 (0.21, 1.85)	0.39	0.34 (0.07–1.54)	0.16
ACUITY bleeding						
Major	334/8064 (4.1)	31/2855 (1.1)	0.25 (0.18, 0.37)	<0.0001		
Minor	879/8064 (10.9)	233/2855 (8.2)	0.72 (0.63, 0.85)	<0.0001		
Major/minor	1173/8064 (14.5)	262/2855 (9.2)	0.60 (0.52, 0.68)	<0.0001	0.70 (0.59–0.83)	<0.0001
All non-CABG bleeding	1173/8064 (14.5)	262/2855 (9.2)	0.60 (0.52, 0.68)	<0.0001		

GUSTO, Global Use of Strategies to Open Occluded Arteries; Thrombolysis In Myocardial Infarction; ACUITY, Acute Catheterization and Urgent Intervention Triage strategY; CABG, coronary artery bypass graft; OR, odds ratio; CI, confidence interval.

## Discussion

Intravenous adenosine diphosphate (ADP) receptor blockade with cangrelor, when compared with clopidogrel, reduced the primary composite outcome of death, MI, IDR, or stent thrombosis at 48 h after randomization regardless of PCI access site. Among the femoral access subjects, cangrelor compared with clopidogrel reduced the odds of the primary composite outcome by 21%; within the radial cohort, there was a consistent 24% reduction in the odds of the primary composite outcome. Although the interaction tests for access site did not reach statistical significance, when the primary composite endpoint was evaluated according to access site, cangrelor demonstrated a reduction in odds of ischaemic events among patients undergoing femoral PCI. In patients undergoing PCI via the radial artery, cangrelor demonstrated a non-significant trend towards fewer ischaemic events, likely due to lack of statistical power because of a smaller sample size. In the femoral cohort, cangrelor's benefit with respect to ischaemic events was driven by a reduction in MI and stent thrombosis, consistent with the overall CHAMPION PHOENIX results. The radial cohort, however, only experienced a reduction in MI. The lack of benefit regarding stent thrombosis in the radial cohort is conceivably due to a low event frequency within this particular subgroup: 95 total (1.2%) in the femoral vs. 25 total (0.9%) in the radial.

In both the femoral and radial groups, cangrelor compared with clopidogrel was not associated with a significant increase in the pre-specified GUSTO-defined severe bleeding, the primary safety endpoint, or in blood transfusions; though more sensitive definitions such as ACUITY-defined bleeding did show increased rates of bleeding with cangrelor in both the femoral and radial cohorts. It is important to note that the absolute rates of GUSTO severe/moderate bleeding and blood transfusions were approximately two to three times higher with the femoral approach compared with the radial, in both the cangrelor and clopidogrel arms of the study. These findings are consistent with prior studies. The analysis of all femoral vs. radial randomized PCI clinical trials has found radial access, when compared with femoral, to be associated with a 42% reduction in non-CABG bleeding.^5^ Contemporary studies have demonstrated that up to 70% of bleeding events occurring in the PCI setting can be attributed to complications arising at the site of vascular access.^12^ The present study suggests that in a large contemporary international trial, the radial approach for PCI has the potential to play a key role in reducing periprocedural bleeding in a wide variety of PCI patients.

It is postulated that a reduction in access site bleeding may in turn lead to fewer subsequent adverse events.^13^ For example, the aforementioned 42% reduction in major non-CABG bleeding associated with the radial approach for PCI was paralleled with an aggregate reduction of major adverse cardiac events (death, MI, or stroke) of 14%, *P* = 0.005.^5^ After multivariable analysis the present study finds the odds of periprocedural bleeding 30–66% lower, depending on bleeding definition, when PCI was performed via the radial artery compared with the femoral approach. However, the favourable bleeding profile associated with the radial artery approach to PCI in CHAMPION PHOENIX did not translate into a reduction in the primary efficacy endpoint at 48 h.

There are certain limitations to this analysis. First, similar to the overall CHAMPION PHOENIX trial, there is the potential for the benefit of cangrelor to be attenuated in the setting of more prolonged pretreatment with clopidogrel or with the use or ticagrelor or prasugrel. Secondly, bleeding endpoints were not adjudicated. Thirdly, the treatment by radial vs. femoral access was not randomized and even with the adjusted analyses, there may be residual confounding. Lastly, CHAMPION PHOENIX was not powered to test the interaction between treatment and PCI access site; therefore, all interaction terms should be interpreted with caution.

In CHAMPION PHOENIX, intravenous ADP-receptor inhibition with cangrelor reduced ischaemic events with no significant increase in severe bleeding or blood transfusions regardless of PCI access site. Compared with the femoral approach, rates of bleeding complications appeared to be lower with radial access for PCI in both randomized arms of the study.

## Authors' contributions

J.P.: performed statistical analysis. D.L.B.: handled funding and supervision, acquired the data, and conceived and designed the research. J.A.G.: drafted the manuscript. R.A.H.: made critical revision of the manuscript for key intellectual content. All authors analyzed the data and interpreted the results. All authors contributed to writing revisions and approved the final manuscript.

## Supplementary material

Supplementary material is available at *European Heart Journal* online.

## Funding

The CHAMPION PHOENIX trial was funded by The Medicines Company.

**Conflict of interest:** J.A.G. discloses the following relationships—Honoraria: Boehringer Ingelheim. R.A.H. discloses the following relationships—Advisory Board: Evidint, Regado, Scanadu; Honoraria: Amgen, Daiichi-Lilly, Gilead Sciences, Gilead Sciences, Inc., Janssen R and D, Medtronic, Merck, Novartis Corporation, The Medicines Company, Vida Health, Vox Media, WebMD; Other: American Heart Association; Research Funding: AstraZeneca, BMS, CSL Behring, GSK, Merck, Portola, Sanofi-Aventis, The Medicines Company; Ownership Interest: Element Science, MyoKardia. J.C.B. has no conflict of interests to declare. G.W.S. discloses the following relationships—Honoraria: Boston Scientific, InspireMD, Atriaum, Eli Lilly—Daiichi Sankyo partnership, AstraZeneca. Ph.G.S. discloses the following relationships—Honoraria: Amarin, AstraZeneca, Bayer; Other: The Medicines Company. C.M.G. discloses the following relationships—Honoraria: The Medicines Company. C.W.H. discloses the following relationships—Honoraria: AstraZeneca, Sanofi-Aventis, Lilly; Research Funding: Astra Zeneca, The Medicines Company. M.J.P. discloses the following relationships—Honoraria: AstraZeneca, Merck & Co., Accriva Diagnostics, The Medicines Company. P.G. discloses the following relationships—Honoraria: Abbott Vascular. J.P. discloses the following relationships—Employment: The Medicines Company. E.N.D. discloses the following relationships—Employment: The Medicines Company. K.W.M. discloses the following relationships—Honoraria: Bayer, Boehringer Ingelheim, Bristol-Myers Squibb, Cubist, Eli Lilly, Epson, Forest, Glaxo Smith Kline, Johnson & Johnson, Medtronic, Merck, Mt. Sinai, Myokardia, Omthera, Portola, Purdue Pharma, Spring Publishing, Vindico, WebMD; Research Funding: Daiichi, Johnson & Johnson, Medtronic, St. Jude, Tenax. H.D.W. discloses the following relationships—Honoraria: AstraZeneca; Research Funding: Sanofi-Aventis, Eli Lilly, National Health Institute, Glaxo Smith Kline, Merck Sharpe & Dohme, AstraZeneca. D.L.B. discloses the following relationships—Advisory Board: Cardax, Elsevier Practice Update Cardiology, Medscape Cardiology, Regado Biosciences; Board of Directors: Boston VA Research Institute, Society of Cardiovascular Patient Care; Chair: American Heart Association Get With The Guidelines Steering Committee; Data Monitoring Committees: Duke Clinical Research Institute, Harvard Clinical Research Institute, Mayo Clinic, Population Health Research Institute; Honoraria: American College of Cardiology (Senior Associate Editor, Clinical Trials and News, ACC.org), Belvoir Publications (Editor in Chief, Harvard Heart Letter), Duke Clinical Research Institute (clinical trial steering committees), Harvard Clinical Research Institute (clinical trial steering committee), HMP Communications (Editor in Chief, Journal of Invasive Cardiology), Journal of the American College of Cardiology (Guest Editor; Associate Editor), Population Health Research Institute (clinical trial steering committee), Slack Publications (Chief Medical Editor, Cardiology Today's Intervention), WebMD (CME steering committees); Other: Clinical Cardiology (Deputy Editor); Research Funding: Amarin, AstraZeneca, Bristol-Myers Squibb, Eisai, Ethicon, Forest Laboratories, Ischemix, Medtronic, Pfizer, Roche, Sanofi-Aventis, The Medicines Company (including for his role as Co-Chair of CHAMPION PHOENIX); Site Co-Investigator: Biotronik, St. Jude Medical; Trustee: American College of Cardiology; Unfunded Research: FlowCo, PLx Pharma, Takeda.

## Supplementary Material

Supplementary Data
