# Supplementary material for: The effect of cangrelor and access site on ischaemic and bleeding events: insights from CHAMPION PHOENIX
Source: Eur Heart J. 2015 Sep 23;37(14):1122–30. doi: 10.1093/eurheartj/ehv498 (PMC4823635; doi:10.1093/eurheartj/ehv498)
Supplement: Supplementary Data [file ehv498_supplementary_data.zip › ehv498supp.docx]

**Supplementary Appendix**

This appendix has been provided by the authors to give readers additional information about their work.

Supplement to:

The Effect of Cangrelor and Access Site on Ischemic and Bleeding Events – Insights from CHAMPION PHOENIX

J. Antonio Gutierrez, MD MHS, Robert A. Harrington, MD, James C. Blankenship, MD, Gregg W. Stone, MD, Ph. Gabriel Steg, MD, C. Michael Gibson, MS, MD, Christian W. Hamm, MD, Matthew J. Price, MD, Philippe Genereux, MD, Jayne Prats, PhD, Efthymios N. Deliargyris, MD, Kenneth W. Mahaffey, MD, Harvey D. White, DSc, Deepak L. Bhatt, MD, MPH, on Behalf of the CHAMPION PHOENIX Investigators*

*A full list of investigators can be found in Bhatt DL et al. NEJM 368(14):1303-13, 2013.

**The Effect of Cangrelor and Access Site on Ischemic and Bleeding Events – Insights from CHAMPION PHOENIX Supplementary Materials**

**Contents**

**Table S1: Baseline characteristics: radial vs. femoral ………………………………….……3**

**Table S2: Procedure characteristics: radial vs. femoral……………………………………...4**

**Table S3: Efficacy endpoints at 48 hours: radial vs. femoral……………………..…………5**

| Table S1. Baseline characteristics: radial vs. femoral. | | | |
| --- | --- | --- | --- |
|  | **Femoral** | **Radial** | **P-value** |
| Characteristic |  |  |  |
| Demographic |  |  |  |
|  | N=8064 | N=2855 |  |
| Age-years |  |  |  |
| Median | 64 | 64 | 0.28 |
| Interquartile range | 56, 72 | 56, 72 |  |
| Female sex, *n* (%) | 2251 (27.9) | 795 (27.8) | 0.94 |
| Weight - kilograms |  |  |  |
| Median | 84 | 84 | 0.004 |
| Interquartile range | 73, 96 | 74, 96 |  |
|  |  |  |  |
| Medical history, *n* (%) | | | |
| Diabetes mellitus | 2200 (27.3) | 847 (29.7) | 0.01 |
| Current smoker | 2290 (29.1) | 755 (27.1) | 0.04 |
| Hypertension | 6395 (79.6) | 2291 (80.3) | 0.38 |
| Hyperlipidemia | 4800 (69.0) | 1886 (69.5) | 0.62 |
| Prior stroke or TIA | 390 (4.9) | 122 (4.3) | 0.22 |
| Prior myocardial infarction | 1758 (21.9) | 503 (17.7) | <0.0001 |
| Prior PTCA or PCI | 1860 (23.1) | 736 (25.8) | 0.004 |
| CABG | 931 (11.6) | 144 (5.0) | <0.0001 |
| Heart failure | 855 (10.6) | 277 (9.7) | 0.18 |
| Peripheral artery disease | 596 (7.5) | 231 (8.2) | 0.19 |

TIA = transient ischemic attack, PTCA = percutaneous transluminal coronary angioplasty, PCI = percutaneous coronary intervention, CABG = coronary artery bypass graft

| Table S2. Procedure characteristics: radial vs. femoral. | | | |
| --- | --- | --- | --- |
| Characteristic | **Femoral** | **Radial** | **P-value** |
|  | N=8064 | N=2855 |  |
| Indication*, n* (%) |  |  |  |
| Stable angina | 4548 (56.4) | 1793 (62.8) | <0.0001 |
| NSTE ACS | 2184 (27.1) | 706 (24.7) |  |
| STEMI | 1332 (16.5) | 356 (12.5) |  |
|  |  |  |  |
| Periprocedural antithrombotic, *n* (%) |  |  |  |
| Aspirin | 7647/8057 (94.9) | 2642/2854 (92.6) | <0.0001 |
| Clopidogrel, 300 mg loading dose  (planned) | 2654 (32.9) | 141 (4.9) | <0.0001 |
| Clopidogrel, 600 mg loading dose  (planned) | 5410 (67.1) | 2714 (95.1) | <0.0001 |
| Low-molecular-weight heparin | 1159/8064 (14.4) | 324/2853 (11.4) | <0.0001 |
| Unfractionated heparin | 6065/8063 (75.2) | 2463/2855 (86.3) | <0.0001 |
| Fondaparinux | 209/8064 (2.6) | 82/2854 (2.9) | 0.42 |
| Bivalirudin | 1884/8062 (23.4) | 633/2855 (22.2) | 0.19 |
| Glycoprotein IIb/IIIa inhibitor | 284 (3.5) | 95 (3.3) | 0.63 |
|  |  |  |  |
| Duration of PCI - minutes | N=8064 | N=2854 | 0.34 |
| Median | 17 | 18 |  |
| Interquartile range | 10, 30 | 10, 30 |  |
| Drug-eluting stent, *n* (%) | 4313 (53.5) | 1763 (61.8) | <0.001 |
| Bare-metal stent, *n* (%) | 3613 (44.8) | 1025 (35.9) | <0.001 |
| Balloon angioplasty, *n* (%) | 394 (4.9) | 169 (5.9) | 0.03 |
|  |  |  |  |
| PCI success, *n* (%) | 7916 (98.2) | 2806 (98.3) | 0.68 |

NSTE ACS = non ST elevation acute coronary syndrome, STE ACS = ST elevation acute coronary syndrome, PCI = percutaneous coronary intervention

| Table S3. Efficacy at 48 hours: radial vs. femoral. | | | | | | |
| --- | --- | --- | --- | --- | --- | --- |
| Endpoint, *n/N* (%) | **Femoral** | **Radial** | **OR (95% CI)**  **Unadjusted** | **P-value**  **Unadjusted** | **OR (95% CI)**  **Adjusted** | **P-value**  **Adjusted** |
|  |  |  |  |  |  |  |
| Death/MI/IDR/ST | 432/8064 (5.4) | 145/2852 (5.1) | 0.95(0.78,1.15) | 0.58 | 1.03(0.81,1.29) | 0.83 |
| Death | 29/8064 (0.4) | 7/2852 (0.2) | 0.68(0.30,1.56) | 0.36 | 0.98(0.37,2.58) | 0.96 |
| MI | 333/8064 (4.1) | 127/2852 (4.5) | 1.08(0.88,1.33) | 0.46 | 1.13(0.88,1.45) | 0.34 |
| IDR | 48/8064 (0.6) | 18/2852 (0.6) | 1.06(0.62,1.83) | 0.83 | 1.29(0.67,2.46) | 0.44 |
| ST | 95/8064 (1.2) | 25/2852 (0.9) | 0.74(0.48,1.15) | 0.18 | 0.93(0.55,1.56) | 0.78 |

MI = myocardial infarction, IDR = ischemia-driven revascularization, ST = stent thrombosis,

OR = odds ratio, CI = confidence interval
